# *TERT* genetic variability and telomere length as factors affecting survival and risk in acute myeloid leukaemia

**DOI:** 10.1038/s41598-021-02767-1

**Published:** 2021-12-02

**Authors:** Marta Dratwa, Barbara Wysoczańska, Aleksandra Butrym, Piotr Łacina, Grzegorz Mazur, Katarzyna Bogunia-Kubik

**Affiliations:** 1 Laboratory of Clinical Immunogenetics and Pharmacogenetics, Hirszfeld Institute of Immunology and Experimental Therapy, Polish Academy of Sciences, Wrocław, Poland; 2 Department of Cancer Prevention and Therapy, Wroclaw Medical University, Wrocław, Poland; 3 Department of Internal, Occupational Diseases, Hypertension and Clinical Oncology, Wroclaw Medical University, Wrocław, Poland

**Keywords:** Cancer genetics, Haematological cancer

## Abstract

Acute myeloid leukaemia (AML) is a neoplasm of immature myeloid cells characterized by various cytogenetic alterations. The present study showed that in addition to the *FLT3*-ITD and *NPM1* mutation status, telomere length (TL) and telomerase reverse transcriptase (*TERT*) gene polymorphisms may affect risk and overall survival (OS) in AML. TL was longer in healthy controls than in AML patients and positively correlated with age in the patients, but not in healthy subjects. TL was found to be independently affected by the presence of the *FLT3*-ITD mutation. As for the *TERT* gene polymorphism, AML patients with the *TERT* rs2853669 *CC* genotype were characterized by significantly shorter OS than patients carrying the *T* allele. Another observation in our study is the difference in TL and OS in patients belonging to various risk stratification groups related to the *FLT3*-ITD and *NPM1* mutation status. Patients with adverse risk classification (mutation in *FLT3*-ITD and lack of mutation in *NPM1*) presented with the shortest telomeres and significantly worse OS. In conclusion, OS of AML patients appears to be affected by *TERT* gene variability and TL in addition to other well-established factors such as age, WBC count, or *FLT3*-ITD and *NPM1* mutation status.

## Introduction

Acute myeloid leukaemia (AML) is a heterogeneous haematological malignancy, characterized by clonal expansion of abnormal immature leukaemic blasts^1–3^. Molecular changes in driver genes, polymorphic abnormalities and coexisting common mutational spectra occurring in AML are important prognostic and predictive markers in younger as well as in older AML patients^4–6^. Basic risk stratification and prognostic scoring of AML is based on the presence of mutation within nucleophosmin member 1 gene (*NPM1*) and/or signal transduction fms-like tyrosine kinase 3 (*FLT3*) gene, and groups patients into favourable, intermediate, and adverse risk categories^7^. There are many other gene mutations described in AML that are stratified according to the different functional pathways in which these genes are involved (e.g., oncogenes, transcription factors, tumour suppressors, epigenetic and chromatin modifying genes), most of which are important in diagnostics and modern therapy of AML patients^8^.

The *FLT3*-internal tandem duplication (*FLT3*-ITD) is the most common genetic alteration and is identified in approximately 25% of AML patients^9,10^. It leads to proliferative activation by continuous phosphorylation of the FLT3 receptor, and at the same time suppresses apoptosis. In clinical practice, *FLT3*-ITD mutations are independent markers of poor prognosis in cytogenetically normal AML^10^. Moreover, they are associated with an aggressive disease phenotype and shorter overall survival^11^. Another important gene abnormality identified in both young and older AML patients is the *NPM1* mutation^12^. This mutation is found in almost one-third of newly diagnosed cases and leads to mislocalized NPM1 protein, found in the cytoplasm instead of the nucleolus^13^. In the absence of the *FLT3*-ITD alteration, *NPM1* mutation is associated with a favourable prognosis and possibility for complete remission of the disease in AML patients^14^.

AML it the most common haematological neoplasm associated with short telomeres^15^. The occurrence of telomere shortening in leukaemias depends particularly on telomerase activity, telomerase reverse transcriptase catalytic subunit (*TERT*) expression, *TERT* promoter gene mutation (*TERT*p), and variability within the *TERT* gene^16–19^. Reduction of telomerase activity and extremely short telomeres induce chromosomal instability, causing bone marrow failure. *TERT* overexpression is observed in 80–95% of malignant cells and this dysregulation in the cancer cells can be explained by factors that lead to modification e.g. in the *TERT* promotor structure^20^.

A better understanding of the molecular mechanisms underlying AML led to development of drugs and new treatment strategies^21^. Standard clinical treatment of AML patients consists of high-intensity induction chemotherapy and/or haematopoietic stem cell transplantation^22^. However, many newly diagnosed AML patients do not qualify for intensive chemotherapy because of their age (> 75 years) or comorbidities^23,24^. Moreover, patients with *FLT3* mutations are characterized with a much worse response to chemotherapy. Nowadays, therapies using various FLT3 tyrosine kinase inhibitors are applied in AML patients with *FLT3* mutation^24^. Unfortunately, effective treatment of AML patients is challenging because of a very clonal heterogeneity of the disease and the occurrence of drug resistance.

In the present study we aimed to analyse AML patients in terms of the presence of *FLT3-*ITD and/or *NPM1* gene mutations, telomere length and genetic variability within catalytic subunit of telomerase (*TERT*) in younger and old AML patients with respect to the clinical data, including overall survival (OS).

## Results

### Telomere length in patients and controls

**Figure 1:**
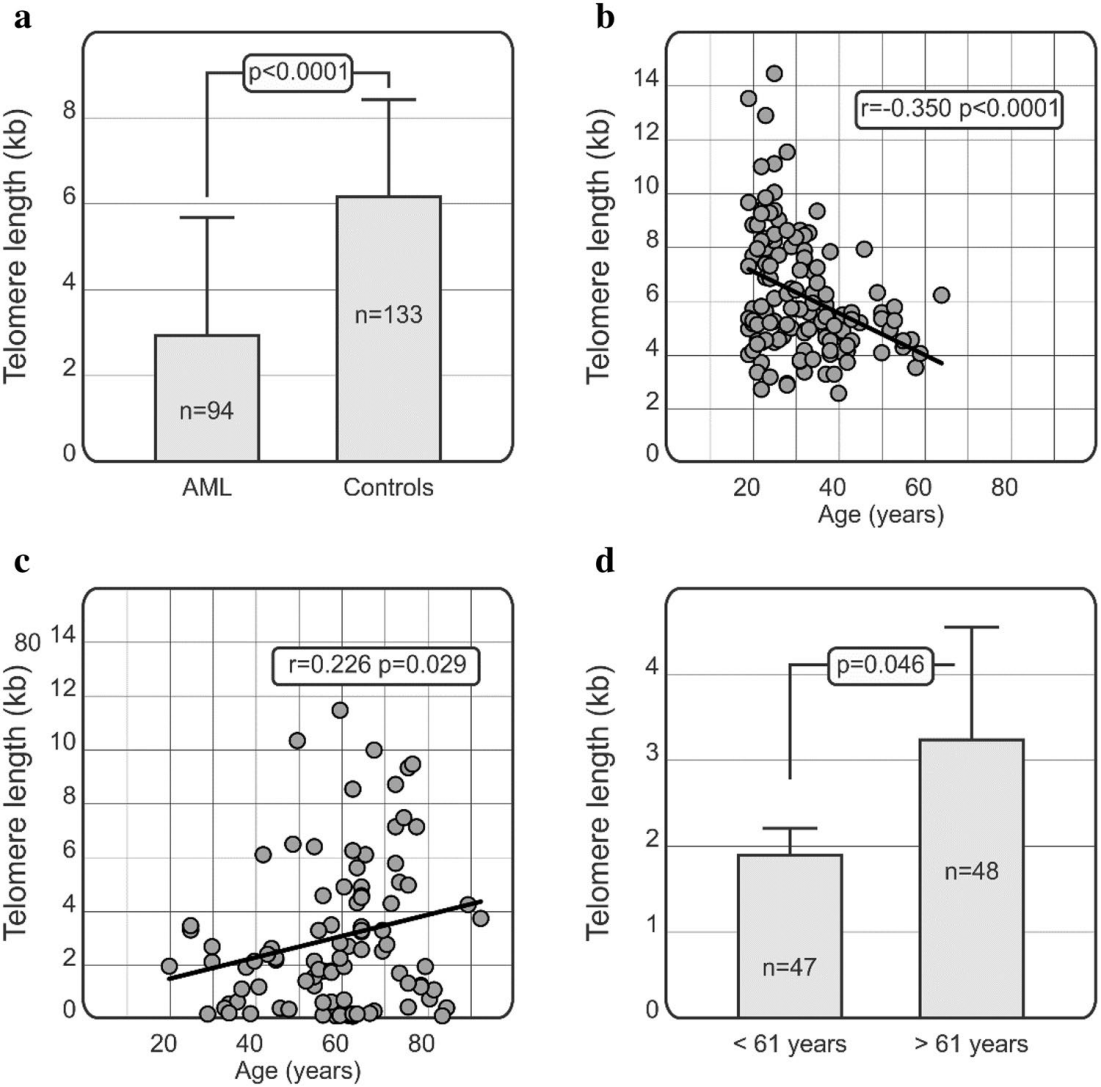
Comparison of telomere length in AML patients and controls (**a**) and relationships between age and telomere length in patients and controls. Telomere length correlates negatively with age in healthy controls (**b**) but not in AML patients (**c**). Statistical analysis was performed using Pearson correlation (PC) tests. Comparison of telomere length in AML patients below and above 61 years of age. Mann-Whitney U test was employed to assess the significance of differences in telomere length (**d**).

A significant difference (p < 0.0001) was observed between telomere length of healthy controls and AML patients (Fig. 1a), this was confirmed in an age-adjusted logistical regression analysis (p < 0.0001). In healthy controls, mean telomere length equalled 6.16 ± 2.27 kb (median: 5.56 kb, range from 2.58 kb to 14.43 kb) while in AML patients telomere length was shorter and equalled 2.94 ± 2.75 kb (median 2.09 kb, range from 0.02 kb to 11.48 kb). Moreover, telomere length declined with age in healthy subjects (r = − 0.350, p < 0.0001), while it increased in AML cases (r = 0.226, p = 0.029) (Fig. 1b,c).

When the patients were subdivided with respect to the median age at diagnosis (61 years), it was observed that the older patients presented with longer telomeres as compared to younger patients (3.33 ± 2.84 vs 1.89 ± 3.73 kb, p = 0.046; Fig. 1d). There were 47 patients younger than 61 years and 48 older than 61 years.

### Effect of the TERT gene polymorphism on overall survival

We did not observe any statistically significant differences in allele and genotype distribution between AML patients and healthy individuals for any of the *TERT* SNPs (rs2736100, rs2853669) studied (Table 1). Thus, in our AML patients, none of *TERT* (rs2736100, rs2853669) variants was found to affect the susceptibility of the disease.

**Table 1:** Distribution of *TERT* genotypes in acute myeloid leukaemia (AML) patients and healthy individuals.

	AML patients (n = 91)	Healthy individuals (n = 133)
***TERT* rs2736100 (intron 2)**		
*CC*	21 (23.1%)	31 (23.3%)
*AC*	47 (51.6%)	58 (43.6%)
*AA*	23 (25.3%)	44 (33.1%)
***TERTp* rs2853669 (promoter)**		
*TT*	58 (63.7%)	81 (61%)
*CT*	24 (26.4%)	43 (32%)
*CC*	9 (9.9%)	9 (7%)

**Figure 2:**
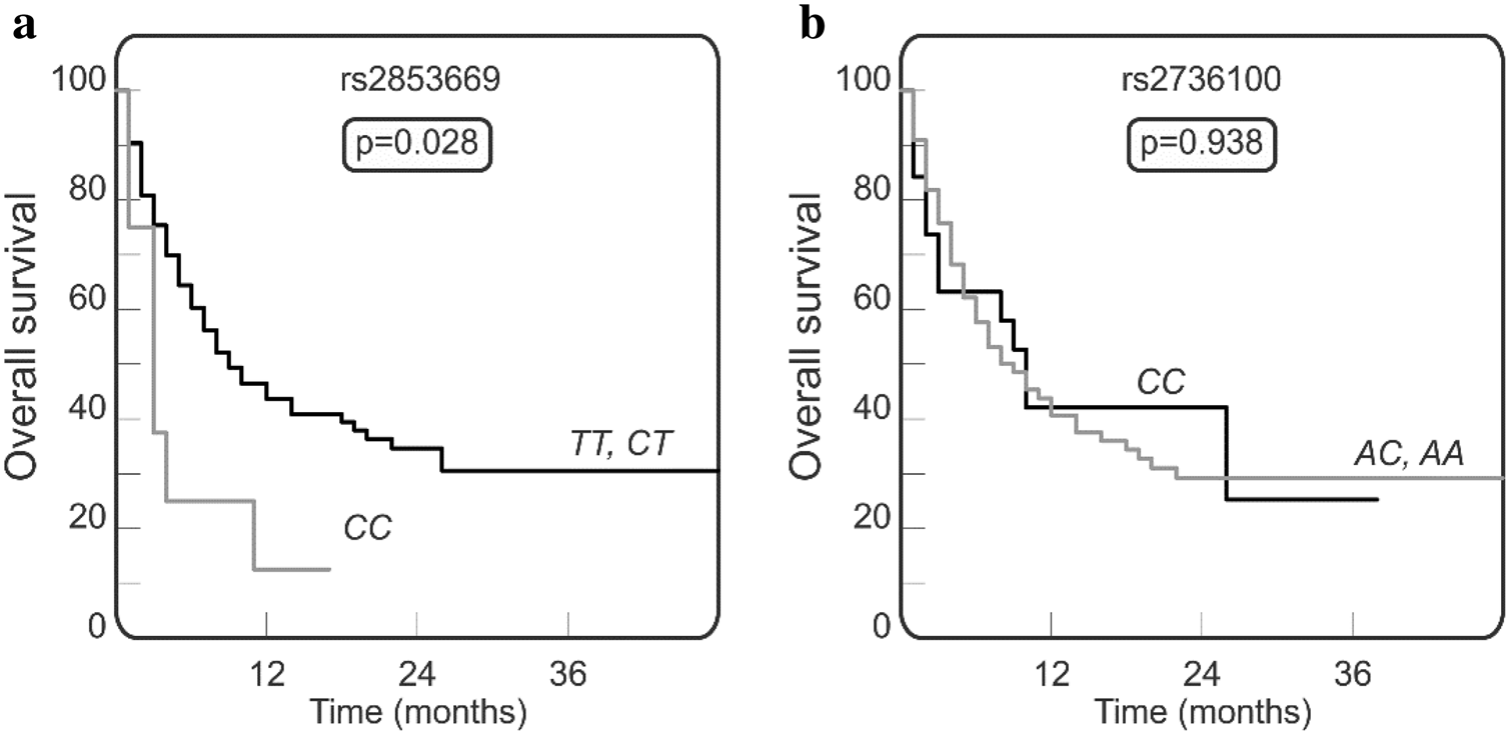
Overall survival in AML patients carrying various genotypes of *TERT* rs2853669 (**a**) and *TERT* rs2736100 (**b**). The homozygous rs2853669 *CC* genotype is associated with shorter overall survival (**a**). The Gehan-Breslow-Wilcoxon test was used for statistical analysis.

Employing Kaplan-Meier curves, we compared the overall survival of AML patients carrying various *TERT* genotypes. Patients with rs2853669 *CC* homozygous genotype presented with a shorter overall survival than patients having the rs2853669 *T* allele (carriers of *TT* or *CT* genotypes; p = 0.028; Fig. 2a). The most favourable effect on survival was observed for the rs2853669 *CT* heterozygosity (p = 0.089; not shown). As for the second *TERT* SNP (rs2736100) investigated, no significant association between the presence of any of its genetic variants and overall survival was observed (p = 0.961; Fig. 2b), although there was a trend towards better OS in elderly patients over 61 years old carrying the rs2736100 *CC* genotype (p = 0.051).

### *FLT3*-ITD and/or *NPM1* mutation status in relation to telomere length, overall survival and other clinical parameters

As expected, analysis of the survival curves showed that younger patients (below median age of 61 years) lived longer than the older patients with a median overall survival of 12 and 5 months, respectively (p = 0.007).

**Figure 3:**
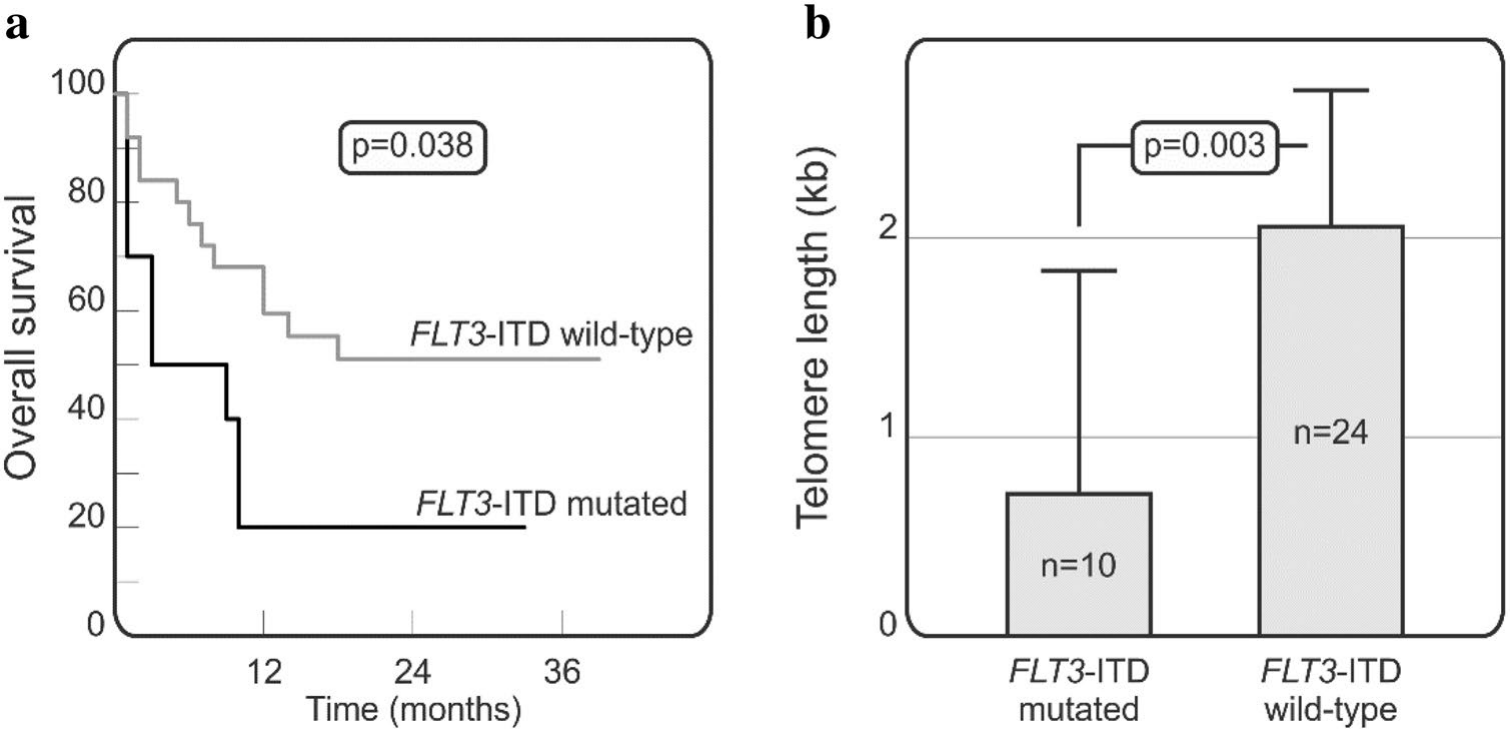
The effect of the presence of *FLT3*-ITD mutation on telomere length and survival in patients below 61 years of age. Kaplan-Meier curves for overall survival in patients stratified with respect to the presence of *FLT3*-ITD mutation. Gehan-Breslow-Wilcoxon test was used for statistical analysis (**a**). Differences in median telomere length in AML patients having or lacking *FLT3*-ITD mutation assessed by Mann-Whitney U test (**b**).

Interestingly, some additional associations related to the presence of the unfavourable *FLT3*-ITD mutation were noted in the group of patients below 61 years of age. We observed, that among AML patients below 61 years, those carrying the *FLT3*-ITD mutation had significantly lower median telomere length 0.72 ± 0.81 kb (range from 0.02 to 2.19 kb) when compared to *FLT3*-ITD wild type cases with median telomere length of 2.07 ± 2.30 kb (range from 0.1 to 10.35 kb) (p = 0.003; Fig. 3a). Moreover, the presence of *FLT3*-ITD mutation in this group of AML patients was found to be associated with significantly worse overall survival (p = 0.038; Fig. 3b). On the other hand, no significant relationships were observed for *NPM1* mutation.

Multivariate Cox regression analysis was employed to confirm independent associations of selected parameters with overall survival of AML patients. The following factors were considered: age, white blood cell (WBC) count, *FLT3*-ITD and *NPM1* mutation status (mutated vs wild-type), telomere length as well as *TERT* rs2853669 (*CT* v*s CC* + *TT*) polymorphism. This analysis demonstrated that variability within the *TERT* gene and mutation status may influence overall survival. The analysis documented that rs2853669 heterozygosity (p = 0.0557) and the presence of *NPM1* mutation (p = 0.0827) showed a positive association with overall survival, while higher WBC count (p = 0.0002) and more advanced age (p = 0.0448) showed an adverse effect (Table 2).

**Table 2:** Multivariate analysis of factors potentially affecting overall survival in patients with AML.

	HR (95% CI)	*p*-value
Age	1.054 (1.001–1.109)	0.0448
WBC	1.010 (1.004–1.014)	0.0002
*FLT3*-ITD, mutated vs wild-type	2.854 (0.826–9.866)	0.0975
*NPM1*, mutated vs wild-type	0.256 (0.055–1.193)	0.0827
Telomere length	0.709 (0.455–1.105)	0.1291
*TERT* rs2853669 (*CT* vs *CC* + *TT*)	0.324 (0.102–1.024)	0.0557

*HR* Hazard ratio, *CI* confidence interval, *WBC* white blood cells count, *FLT3-ITD* internal tandem duplication of the *FLT3* gene, *NPM1* nucleophosmin member 1 gene.

Additional multivariate Cox regression analysis including age, WBC count, *FLT3*-ITD and *NPM1* mutation status (mutated vs wild-type), as well as *TERT* rs2853669 (*CC* v*s CT* + *TT*) homozygosity showed that *CC* genotype was also associated with overall survival in AML patients (HR 8.066, p = 0.0230). As expected, the higher WBC count (HR 1.009, p < 0.0001) and advanced age (HR 1.053, p = 0.0352) showed a negative impact on overall survival. This analysis also confirmed that the presence of the *FLT3*-ITD (HR 3.518, p = 0.0410) and *NPM1* (HR 0.272, p = 0.0680) mutations influence overall survival in an opposite way.

To assess whether the *FLT3*-ITD and/or *NPM1* mutation status could act as an independent risk factor affecting telomere length in AML patients, a multiple linear regression model considering the presence of the *FLT3*-ITD and *NPM1* mutation and both SNPs was employed. It was found that the presence of *FLT3*-ITD significantly affected telomere length that it was shorter in the *FLT3*-ITD positive cases (p = 0.002), while patients positive for the *NPM1* mutations tended to have longer telomeres; p = 0.074). In this analysis, none of the two SNPs appeared to be an independent factor for telomere length.

As for the associations with other clinical parameters, we observed that patients positive for *FLT3*-ITD mutation showed increased lactate dehydrogenase (LDH) levels (with an average of 763.5 U/l; *p* = 0.048) and a tendency towards a higher WBC count (112.5 × 10^9^/l; *p* = 0.072).

**Figure 4:**
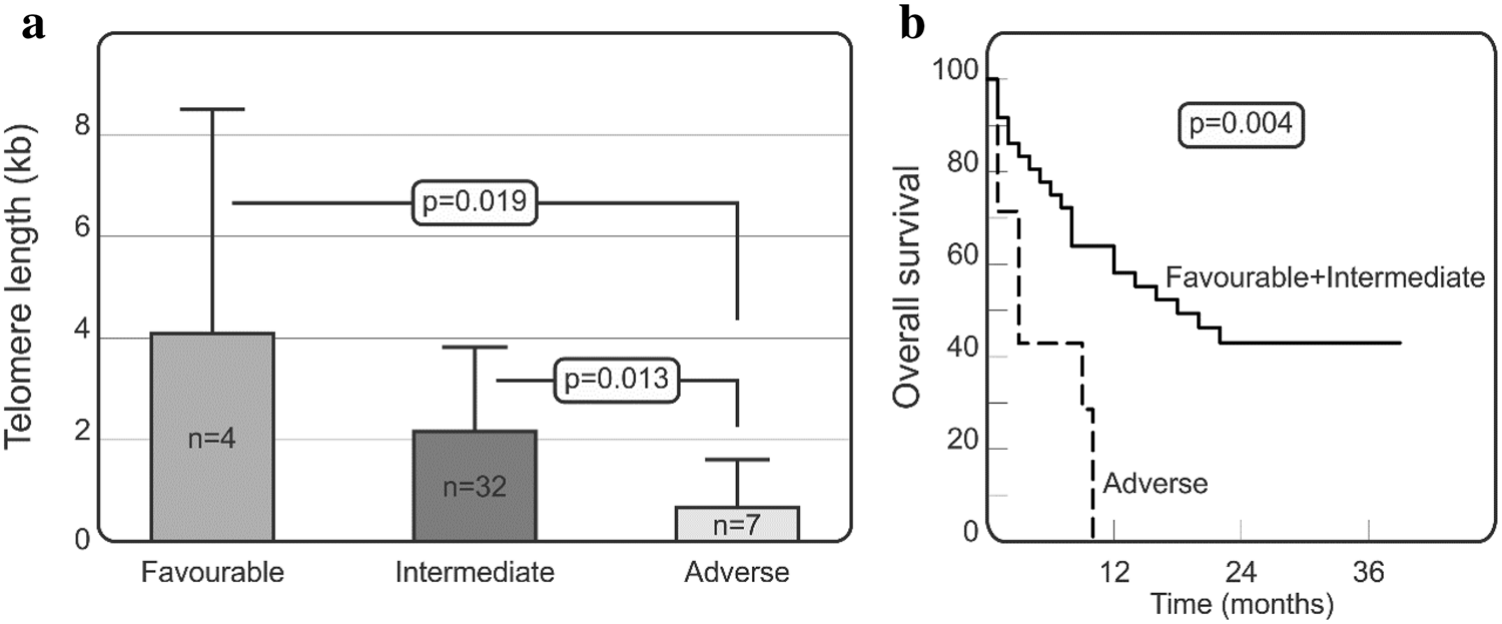
Telomere length and overall survival of patients in various ELN risk groups. Kruskal–Wallis test with the Original FDR method of Benjamini and Hochberg were employed to assess the significance of differences in mean telomere length in AML patients with favourable, intermediate, and adverse risk stratification (**a**). Gehan–Breslow–Wilcoxon test for statistical analysis of overall survival curves in AML patients was performed (**b**).

As the *FLT3*-ITD and *NPM1* mutations are included, among other parameters, in the 2017 European LeukemiaNet (ELN) criteria^7^ of AML patients, we decided to check if telomere length and overall survival differ between patients in ELN risk groups (favourable, intermediate, adverse). As shown in Fig. 4a, AML patients with favourable risk classification characterized with longer telomeres as compared to AML patients with adverse risk. The mean telomere length equalled 4.08 ± 4.39 vs. 0.66 ± 0.94 kb, for patients with favourable and adverse risk respectively, p = 0.019; Fig. 4a). We also observed that patients with intermediate risk classification had longer telomeres as compared to the adverse group (2.15 ± 1.66 vs. 0.66 ± 0.94 kb, p = 0.013; Fig. 4a). Moreover, AML patients with adverse risk were characterized by worse overall survival as compared to patients of the other two risk groups (p = 0.002; Fig. 4b).

## Discussion

Acute myeloid leukaemia arises from the expansion of haematopoietic stem and progenitor cells which acquire numerous somatic mutations^25^. Approximately half of all AML patients are characterized by normal karyotype and have losses or duplications in terminal regions of chromosomes which may affect telomere stability^26^. Moreover, about 40–50 genes were found to harbour recurrent somatic mutations in various AML subtypes^25^. We hypothesised, that telomere length or polymorphisms within the *TERT* gene may also be related to disease risk, and survival.

In the present study we assessed telomere length in healthy blood donors and AML patients. We observed that AML patients were characterized by significantly shorter telomeres than healthy subjects. Similar relationships were shown by Aalbers et al. in the group of children with AML. They observed that telomere length in leukaemic cells was very short as compared to healthy control peripheral blood mononuclear cells^16^. Ventura Ferreira et al. also demonstrated that AML patients were characterized by significantly shorter telomeres than healthy controls^27^. Moreover, in our present study we have also noticed that telomere length decreases with age in healthy controls, but not in AML cases. Previously published data show inconsistent results. For example, a study by Menshawy et al. demonstrated a lack of correlation between age and telomere length in patients with AML^28^. On the contrary, Williams et al. reported a correlation between telomere length and age of diagnosis in AML patients^29^. Interestingly, both studies, our present one and that of Williams et al., report differences in telomere length between patients at different ages. We divided our AML patients into two groups, below and above 61 years of age (reflecting the median age in our group of patients). Our analysis showed that younger patients did have shorter telomere length as compared to older patients. Similar results were reported by Williams et al., who observed that younger AML patients (< 60 years old) had significantly shorter telomeres than patients at a more advanced age^29^.

In several studies, genetic variability within the *TERT* gene was analyzed in order to look for association with predisposition to the disease, its progression/outcome, or survival. We analyzed two *TERT* SNPs, one in the *TERT* promoter region (rs2853669, C/T) and the other located in intron 2 (rs2736100, C/A). We did observe some associations between *TERT* polymorphism and overall survival, although no significant relationship with the risk for the development of AML was found. In our study, patients carrying homozygous rs2853669 *CC* genotype were characterized with shorter overall survival than patients with *T* allele while the *CT* heterozygosity seemed to play more favourable role (see Fig. 3). Our results are consistent with those of Mosrati et al. in Swedish patients with AML. Additionally, Mosrati et al. found an interesting association between *TERT* rs2853669 *CC* homozygosity and increased expression of IL-6 and TNFα, cytokines known as markers for inflammation and cancer progression^19^. Furthermore, they reported that the rs2736100 SNP generated a modest risk for AML, although it had no effect on survival in their AML cohort. The latter observation confirms our results on the lack of association between rs2736100 and survival. On the other hand, it has been reported that in Chinese population, rs2736100 is associated with increased susceptibility to non-small cell lung cancer and myeloproliferative neoplasm^30–32^ as well as AML risk^33^. Tong et al. observed a higher frequency of the *CC* genotype and *C* allele of rs2736100 polymorphism in the Chinese AML patients. However, no significant differences were detected in either genotype or allele distributions between patients and control groups regarding the second SNP (rs2853669)^33^. The above observations may suggest that the effect of *TERT* SNPs may vary between patients from different populations and may be dependent on the broader genetic background of examined populations.

AML is the most common haematologic neoplasm that is associated with short telomeres. Watts et al. suggested that shorter telomere length leads to disconnection of proteins [e.g., telomeric repeat-binding factor 2 (TRF2)] from telomere complex. This biological process may be correlated with loss of major non-telomeric functions such as DNA damage and activation of important repair pathways. Therefore, it seems that telomere length could confer resistance to cytotoxic chemotherapy by affecting DNA repair mechanisms^34^.

Mutations in *FLT3*-ITD and *NPM1* genes are frequently identified in AML, especially in patients with de novo AML and normal karyotype. Both of these genes are involved in important cellular processes, such as differentiation and apoptosis of haematopoietic progenitor cells. The occurrence of mutation in the *NPM1* gene may be beneficial for the health and survival of patients^2^. On the other hand, excessive proliferation and survival of *FLT3*-ITD mutant cell clones have adverse effects for AML patients and is associated with poor prognosis^35^. In our present study, we found some differences between patients having and lacking the *FLT3*-ITD mutation in terms of telomere length and overall survival. Similar relationships were observed also in some previous studies^16,29,34^. Moreover, Molina Garay et al. demonstrated that patients with the *FLT3*-ITD mutation characterized with significantly shorter lifespan as compared to patients lacking the *FLT3*-ITD mutation or with a mutation in the tyrosine kinase domain (*FLT3*-TKD)^36^.

As for the *NPM1* mutation, no association between *NPM1* mutation status and telomere length was found either in our present work or in previous studies^16,29^. However, in our logistic regression model, *NPM1* mutation showed a slight trend towards association with survival.

The novelty of our study is the observation regarding the differences between telomere length in patients belonging to various risk stratification groups (according to the 2017 European LeukemiaNet criteria by Döhner et al.^7^). AML patients carrying *NPM1*, but not *FLT3*-ITD mutation (favourable risk group) were characterized by longer telomeres as compared to AML patients of the adverse risk group (with *FLT*3-ITD mutation and *NPM1* wild-type). Moreover, we observed that patients of the intermediate risk group (*FLT3*-ITD mutation and *NPM1* mutation or *FLT3*-ITD wild-type and *NPM1* wild-type) similarly had significantly longer telomeres in comparison to the adverse risk group of patients. Our AML patients from the adverse risk group were characterized by worse overall survival as compared to patients of the other two risk groups.

Logistic regression analysis showed that WBC count is a strong independent risk factor for survival and the presence of *FLT3*-ITD mutation was associated with higher WBC counts in our AML patients. The latter relationship between WBC count at diagnosis and *FLT3*-ITD mutation status was also reported in a meta-analysis published by Picharski et al.^37^.

In summary, outcomes of patients with the *FLT3*-ITD mutation were significantly worse than of those without this mutation, and higher WBC count was associated with poor prognosis, probably because of the presence of *FLT3* mutation that is likely associated with higher WBC count. We also observed a correlation between LDH levels and *FLT3*-ITD mutation status. Patients positive for *FLT3*-ITD mutation showed higher levels of LDH, suggesting an unfavourable role of LDH in AML patients. In line with this observation, the study of Djunic et al. showed that serum LDH level was the most significant predictor of poor complete remission ratio in AML patients^38^.

In conclusion, overall survival of AML patients depends on various factors such as age, telomere length, mutation status, and *TERT* variability. The presence of *FLT3*-ITD and *NPM1* mutations is used for estimating survival and response to treatment, although new prognostic genetic factors could be used to construct a more detailed risk stratification system. Adverse risk patients (positive for *FLT3*-ITD but negative for *NPM1* mutation) need novel approaches to improve their overall survival and to get a better response to treatment. To clearly demonstrate the role and significance of telomere length in AML patients with normal karyotype, a clinical study involving a larger number of patients may be needed. It seems necessary to explore the new genetic and environmental factors that could be involved in leukaemogenesis.

## Methods

### Characteristics of the study groups

The study involved 95 Polish patients diagnosed with de novo AML with normal karyotype (57 males and 38 females, age range 20–93, median 61 years). Blood samples were collected at diagnosis after obtaining informed consent from patients. All methods were according to the Declaration of Helsinki. Approval of the Bioethical Committee of Wroclaw Medical University was obtained for the study (No. KB-368/2019). WBC count range was 0.7–510.5 × 10^9^/l (median = 21.5 × 10^9^/l). Risk stratification groups included 11% patients with favourable risk, 49% patients with intermediate risk and 40% patients with adverse risk according to 2017 European LeukemiaNet criteria^7^. There were 11 AML patients with *FLT3*-ITD mutation and 34 without it (n = 45). Additionally, there were 8 patients with *NPM1* mutation and remaining 35 without it (n = 43). The median overall survival was 9 months (range 1–122 months). Additionally, 133 blood donors (84 males and 49 females, age range 19–64, median 30 years) served as a control group for the *TERT* polymorphisms and telomere length studies. Due to difference between patients and controls, an age adjusted logistic regression analysis was additionally performed.

### DNA extraction

Genomic DNA was isolated from 10 mL of peripheral blood taken on EDTA using the Qiagen DNA Isolation Kit (Qiagen, Hilden, Germany) following the recommendation of the manufacturer. DNA concentration and purity were quantified on DeNovix (DeNovix Inc., USA). Isolated DNA was used to for *TERT* genotyping and assessment of the telomere length in AML patients.

### Genotyping of *TERT* gene polymorphisms

The selection of investigated single nucleotide polymorphisms (SNPs) within the *TERT* gene was based on results from the SNP Function Prediction tool of the National Institute of Environmental Health Sciences (NCBI Database) website and other auxiliary databases (https://snpinfo.niehs.nih.gov/snpinfo/snpfunc.html; https://www.ncbi.nlm.nih.gov/snp/; https://www.ensembl.org/index.html) the following criteria were used: minor allele frequency in Caucasians above 10%, change in RNA and/or amino acid chain, potential splicing site and/or miRNA binding site.

Based on the above criteria, 2 SNPs were selected for the study. *TERT* rs2736100 (G > T), located in intron 2, is a susceptibility factor for a variety of cancers and myeloproliferative neoplasms. The *TERT* rs2853669 (T > C) SNP located at − 245 bp (Ets2 binding site) in the promoter region, suppresses *TERT* expression and is associated with the enzymatic activity of telomerase. The *TERT* rs2736100 SNP was determined with the use of LightSNiP typing assays (TIB MOLBIOL, Berlin, Germany) while a TaqMan assay was employed for rs2853669 SNP genotyping (ThermoFisher Scientific, USA). Both assays are based on real-time polymerase chain reactions (PCR). Amplifications were performed on a LightCycler480 II Real-Time PCR system (Roche Diagnostics International AG, Rotkreuz, Switzerland) according to the recommendations of the manufacturer. The PCR conditions were as follows: 95 °C for 10 min followed by 45 cycles of: 95 °C for 10 s, 60 °C for 10 s and 72 °C for 15 s. PCR was followed by one cycle of: 95 °C for 30 s, 40 °C for 2 min, and gradual melting from 75 °C to 40 °C.

### Quantification of telomere length

The average telomere length was measured in genomic DNA samples of 91 AML patients and 133 controls. DNA samples were diluted with nuclease-free water to reach a concentration of 5 ng/mL. Telomere length measurements were performed on a LightCycler480 II Real-Time PCR system (Roche Diagnostics International, Rotkreuz, Switzerland) using quantitative polymerase chain reaction (qPCR) assay kits (ScienCell’s Absolute Human Telomere Length Quantification qPCR Assay Kit [AHTLQ], Carlsbad, CA, USA), as previously described^39^. The PCR conditions were as follows: 95 °C for 10 min followed by 32 cycles of: 95 °C for 20 s, 52 °C for 20 s and 72 °C for 45 s. Data analysis was conducted according to manufacturer’s instruction. All reactions were run in three replicates.

### Statistical analysis

The null hypothesis that there is no difference between allele and genotype frequencies between patients and controls was tested with the Fisher's exact test, calculated using the web‐based tool http://vassarstats.net/tab2x2.htm. Survival was assessed using the Gehan-Breslow-Wilcoxon test and Kaplan-Meier survival curves. The remaining statistical analyses of differences between groups were performed using one-way analysis of variance (ANOVA; to determine the significance of differences between the groups), and the resulting p-values were FDR-adjusted using the Benjamini and Hochberg method. For each experiment, data normality was verified with the Shapiro–Wilk test. Considering that distribution of some data deviated from normal distribution, the non-parametric *U* Mann-Whitney test was performed for comparison of telomere length. Statistical calculations were performed by GraphPad Prism software (GraphPad Software, La Jolla, CA, version 8.0.1) and Real Statistics Resource Pack for Microsoft Excel 2019 (version 16.0.10369.20032, Microsoft Corporation, Redmont, Washington, USA). RStudio (RStudio, PBC, Boston, Massachusetts, USA) was used for multiple linear regression model analyses and logistic regression model (Cox regression model) analyses. The r value for the correlation was determined using Pearson correlation (PC) tests. Probability (p) values < 0.05 were considered statistically significant, while those between 0.05 and 0.10 as indicative of a trend. Data in the figures are presented as mean + Standard Deviation (SD) or median + 95% Confidence Interval (CI).

## Data Availability

The datasets generated during and/or analysed during the current study are available from the corresponding author on reasonable request.
